# Exploration of factors associated with spatial−temporal veterinary surveillance diagnoses of rumen fluke (*Calicophoron daubneyi*) infections in ruminants using zero-inflated mixed modelling

**DOI:** 10.1017/S0031182021001761

**Published:** 2022-02

**Authors:** Rhys Aled Jones, Hefin Wyn Williams, Sian Mitchell, Sara Robertson, Michele Macrelli

**Affiliations:** 1 Institute of Biological, Environmental and Rural Sciences, Aberystwyth University, Aberystwyth, UK; 2 Animal and Plant Health Agency (APHA), Job's Well Rd, Carmarthen, SA 31 3EZ, Carmarthenshire, UK; 3 Animal and Plant Health Agency (APHA), Woodham Lane, Addlestone, KT15 3NB, Surrey, UK; 4 Animal and Plant Health Agency (APHA), Rougham Hill, Bury St Edmunds, IP33 2RX, Suffolk, UK

**Keywords:** *Calicophoron daubneyi*, *Fasciola hepatica*, fasciolosis, paramphistomosis, rumen fluke, spatial−temporal modelling, veterinary surveillance, zero inflated mixed models

## Abstract

Rumen fluke (*Calicophoron daubneyi*) has emerged as a prominent parasite of ruminants in Europe over the past decades. Epidemiological questions remain regarding this observed increase in prevalence as well as the prospect for future paramphistomosis risk. This study aimed to identify factors associated with the temporal−spatial prevalence of rumen fluke as measured by veterinary surveillance in a temperate region using zero-inflated negative binomial mixed modelling. Modelling revealed that summer rainfall, raindays and sunshine hours and mean winter temperature as significant positively associated climate variables for rumen fluke prevalence over space and time (*P* < 0.05). Rumen fluke prevalence was also higher in counties with higher cattle/sheep densities and was positively associated with rumen fluke case rates in the previous years (*P* < 0.05). Equivalent models for fasciolosis prevalence revealed no significant association with winter temperature and sunshine hours, (*P* > 0.05). These results confirm a strong association between rainfall and the prevalence of both fluke species in a temperate environment, likely due to the role of *Galba truncatula* as their intermediate snail host. It also highlights the potential added importance of winter temperature and sunshine hours in rumen fluke epidemiology when compared to liver fluke.

## Graphical abstract

**Figure:**
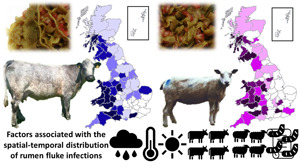

## Introduction

Emerging livestock diseases remain significant threats to sustainable livestock production, animal health and welfare. This threat may be exacerbated in future by climate change and drug resistance (Perry *et al*., 2013). In recent decades, the rumen fluke, *Calicophoron daubneyi*, has emerged as a highly prevalent parasite in cattle herds and sheep flocks across Europe (Arias *et al*., 2011; Jones *et al*., 2017*a*; Ploeger *et al*., 2017; Naranjo-Lucena *et al*., 2018). This sudden appearance is of great concern to farmers and veterinarians due to the potential for heavy juvenile stage infections of *C. daubneyi* in the duodenum to cause clinical disease which in some instances may lead to mortality (Anon, 2018; O'Shaughnessy *et al*., 2018), and the suggestion that adult rumen fluke infecting the rumen may cause sub-clinical disease (Bellet *et al*., 2016; Fenemore *et al*., 2021). This problem is confounded by the lack of basic biological knowledge available regarding rumen fluke epidemiology in temperate countries, a factor that is hampering efforts to successfully assess and combat this threat to animal welfare and productivity (Huson *et al*., 2017). Despite recent molecular confirmation of *C. daubneyi* as the main rumen fluke species present in the UK (Gordon *et al*., 2013) and subsequent clarification of its intermediate snail host species, the mud snail *Galba truncatula* (Jones *et al*., 2015), key epidemiological information regarding *C. daubneyi* remains sparse. These include key factors which will influence *C. daubneyi* transmission, infectiveness and the efficacy of control strategies (Huson *et al*., 2017; Fenemore *et al*., 2021) and furthering our understanding of *C. daubneyi* epidemiology is imperative to minimize paramphistomosis cases in future.

As *C. daubneyi* uses the intermediate snail host *G. truncatula,* it is likely that environmental and climatic factors, which are favourable to *G. truncatula* survival and multiplication, will influence rumen fluke prevalence (Naranjo-Lucena *et al*., 2018). *Galba truncatula* thrives in habitats such as ditches, ponds and boggy areas on pasture, and their populations can multiply rapidly during wet summers (Dreyfuss *et al*., 2015). The relationship between factors that are optimal for *G. truncatula* and *Fasciola hepatica* prevalence, a trematode which also utilizes this snail as an intermediate snail host has been well established (Ollerenshaw, 1971; Smith and Wilson, 1980). Multiple climate models based on these factors, which predicts the suitability of recent climatic conditions on *G. truncatula* presence and population density, and ability to support the parasite's lifecycle have been created to evaluate fasciolosis risk (Ollerenshaw and Rowlands, 1959; Ross, 1975; McIlroy *et al*., 1990). However, despite the utilization of the same intermediate snail host, studies in multiple countries have demonstrated differences between the spatial patterns and infection rates of *C. daubneyi* and *F. hepatica* in cattle and sheep (Szmidt-Adjidé *et al*., 2000; Jones *et al*., 2017*a*; Naranjo-Lucena *et al*., 2018). These differences may be explained by subtle differences between *C. daubneyi* and *F. hepatica* epidemiology outside the main host. These differences include rates of miracidia hatching and cercariae emergence (Titi *et al*., 2014; Chryssafidis *et al*., 2015), metacercariae encystment biology (Abrous *et al*., 2001; Dreyfuss *et al*., 2004) and further unexplored differences which may include egg, miracidia, cercariae and metacercariae survival rates in the environment (Morley, 2011, 2012, 2015; Morley and Lewis, 2017).

To further our understanding of rumen fluke epidemiology, exploratory analyses to identify risk factors associated with the presence and prevalence of rumen fluke across space and time can be conducted through an empirical modelling methodology. Numerous spatial models of rumen fluke prevalence have previously been conducted in multiple European countries (Cringoli *et al*., 2004; Martinez-Ibeas *et al*., 2016; Jones *et al*., 2017*a*; Naranjo-Lucena *et al*., 2018), however models that also incorporate temporal trends are limited. The aim of this study was to record the prevalence of rumen fluke cases as detected by passive veterinary surveillance in cattle and sheep in Great Britain and to identify risk factors associated with the spatial−temporal distribution of these cases. Furthermore, the study aimed to model and identify risk factors of fasciolosis cases detected by veterinary surveillance in cattle and sheep in Great Britain during the same period.

## Methods

### Study area and epidemiological data

This investigation focused on Great Britain which is made up of three countries, England, Scotland and Wales. In 2020, there were 30.7 million sheep and lambs and 8 million cattle and calves present in GB that graze lowland, upland and hill pastures (DEFRA, 2021). The climate of Great Britain is temperate, with average annual temperatures of 10 °C, 7.9 °C and 9.3 °C recorded in England, Scotland and Wales, respectively, between 2010 and 2019 (Hollis *et al*., 2018). Between 2010 and 2019, annual total precipitation amount averages of 865 mm, 1514 mm and 1455 mm were observed in England, Scotland and Wales, respectively (Hollis *et al*., 2018).

This epidemiological study was carried out using the Veterinary Investigation and Diagnosis Analysis (VIDA) databases. The VIDA database contains a record of every diagnostic submission from livestock and wildlife in Great Britain made to the Veterinary Investigation Centres of the Animal and Plant Health Agency (APHA), its partner post-mortem providers, and to Scotland's Rural College (SRUC) Veterinary Services (SRUC VS) and has been operating since 1975. A diagnostic submission is defined by VIDA as a sample or group of samples from one or more animals of the same species, which have been collected within a reasonable time period (e.g. within 48 h), from a farm or premises, in pursuit of a diagnosis of a clinical disease problem. A VIDA diagnosis for rumen fluke and fasciolosis cases is made according to specified diagnostic criteria as seen in Table 1.

**Table 1.** Case definitions of fluke diagnosis categories entered in the VIDA database

Diagnosis category	Definition
Rumen fluke – sheep and cattle	Detection of adult rumen fluke parasites attached to the mucosa of the rumen, or detection of rumen nuke eggs in faeces or detection of immature stages in the proximal small intestine with gross pathology or histological evidence of parasite damage.
Chronic fasciolosis – sheep	Any of the following: (a) demonstration of trematode parasites in liver (b) presence of fluke eggs in faeces (c) positive coproantigen ELISA (d) histopathology, elevated liver enzymes e.g. AST GLDH, GGT and hypalbuminaemia can provide further evidence of ongoing hepatocellular and or chronic biliary damage.
Fasciolosis – cattle	Any of the following: (a) demonstration of *Fasciola* parasites in the liver (b) *Fasciola* eggs in faeces (c) positive coproantigen ELISA (d) histopathology, elevated liver enzymes GLDH, AST & GGT may provide supportive evidence of chronic liver damage. Serology only provides evidence of previous exposure.

A VIDA diagnosis during post mortem examination is not necessarily the cause of death.

According to the VIDA databases, 1994 and 366 cases of rumen fluke were diagnosed in cattle and sheep, respectively, and 5577 and 2796 cases of fasciolosis in cattle and chronic fasciolosis in sheep, respectively were diagnosed in Great Britain (GB) between 2010 and 2019 VIDA diagnoses with no geographical identifier were removed from the dataset prior to analysis. These accounted for 38 and 18 VIDA rumen fluke diagnoses in cattle and sheep respectively, and 75 and 63 diagnoses of fasciolosis in cattle and chronic fasciolosis in sheep, respectively.

### Explanatory variables

Climate variables hypothesized to influence the fluke lifecycle were extracted from the Had-UK-grid database at 5 km resolution (Hollis *et al*., 2018). Annual climate variables averaged across all 5 km grid within individual APHA and SRUC veterinary surveillance counties were used as within-subject independent variables in the model building process. Climate variables used in this study included annual mean summer (April–September) and winter (October–March) temperature, rainfall, raindays and sunshine hours. For each climate variable, long-term averages measured over the 9-year study period were also calculated and incorporated into the model building process as between-subject independent variables. The previous year count of rumen fluke or fasciolosis cases as a proportion of diagnostic submissions was also used as within-subject independent variables in the model building process. The density of cattle (low density – up to 20 cattle km^−2^, medium – between 20 and 40 cattle km^−2^, high – above 40 cattle km^−2^) and sheep (low density – up to 40 sheep km^−2^, medium – between 40 and 80 sheep km^−2^, high – above 80 sheep km^−2^) in each APHA and SRUC surveillance county as categorized by VIDA (APHA, 2021) was incorporated into the model building process as between-subject independent variables.

### Statistical analysis

Negative binomial zero-inflated mixed models were created using the glmmTMB function in R (Brooks *et al*., 2017) to identify factors associated with the absence and number of rumen fluke and fasciolosis cases diagnosed in cattle and sheep. Zero-inflated models account for the presence of excessive zeroes in the dependent variable as was the case in this dataset where no rumen fluke or fasciolosis cases were detected in some surveillance counties during some years. A zero-inflated model calculates the probability of observing a zero count and a negative binomial count value in two separate processes, and thus outputs variables significantly associated with both the absence of VIDA diagnoses (zero-inflated component) and the number of VIDA diagnoses (conditional component) for each respective fluke species (Brooks *et al*., 2017).

VIDA diagnoses of each fluke in each veterinary surveillance county per year were the dependent variable. Years were aligned to the traditional fluke lifecycle in a temperate climate, allowing a lag for maturation of infective fluke to occur permitting for the diagnosis of chronic cases. This meant that each year started in October and finished in September. However, due to the multi-year lifespan of both rumen and liver fluke, it is impossible to specify whether infections had occurred in previous years. County was included as a random factor in the conditional component of each model but was not added as a random factor in the zero-inflated component as this disrupted successful model convergence. No covariance structure was specified in the models as no temporal autocorrelation of residuals was present in any final model. To account for differences in the number of veterinary diagnostic submissions processed, the log number of either cattle or sheep veterinary diagnostic submissions presented in each county per year was added to the models as an offset. Counties averaging less than 20 diagnostic submissions per year were not included in the analysis as their inclusion disrupted model convergence. Therefore, cattle models were based on data from 65 APHA and SRUC surveillance counties (Eileanan an lar, Greater London and Tyne and Wear not included) whilst sheep models were based on data from 60 APHA and SRUC surveillance counties (Bedfordshire, Berkshire, Greater Manchester, Merseyside and West Midlands also not included). A backward elimination method was used to create candidate best-fit models predicting rumen or liver fluke case counts. Here, independent variables were sequentially removed from each model based on their *P* values, with the independent variable with the highest *P* removed before the model was run again. Candidate models were finalized when all variables were significant (*P* < 0.05). Further manual additions and subtractions of independent variables were made in an attempt to improve model fit further.

The candidate models created were tested for their goodness of fit by analysing AIC values, with the models with the lowest AIC regarded as having the best fit. These best fit models were then validated using residual diagnostic tests in the DHARMa package in R (Hartig, 2019). The tests conducted included a test for normality of scaled residuals, a test of deviations between the scaled residuals and rank transformed predictions, spatial and temporal autocorrelation of residuals and zero inflation of residuals. Independent variable multicollinearity and the adjusted intra-class correlation coefficient (ICC) of each model were also calculated using the performance package in R (Lüdecke *et al*., 2021).

## Results

**Fig. 1.**
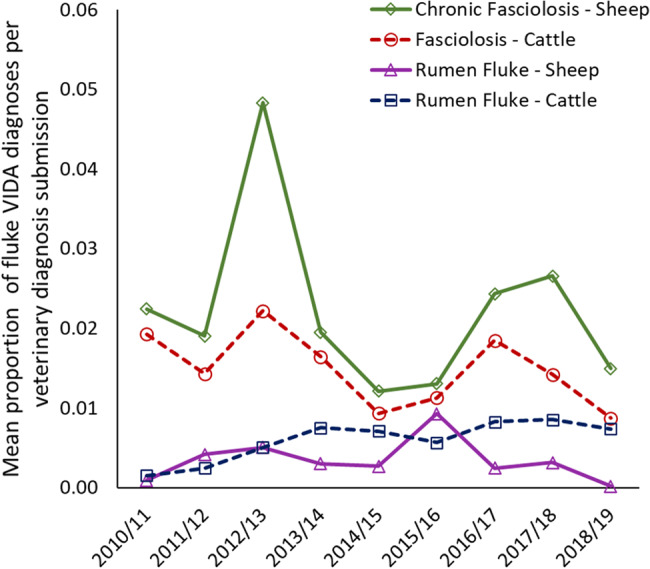
Mean proportion of VIDA fluke diagnoses cases per veterinary diagnostic submission across 67 veterinary surveillance areas in Great Britain between 2010 and 2019.

Figure 1 shows the proportion VIDA diagnosis of rumen fluke, chronic fasciolosis in sheep and fasciolosis in cattle per veterinary diagnostic submission on average in each veterinary surveillance county in Great Britain between 2010 and 2019. Rumen fluke prevalence in cattle and sheep increased between 2010/2011 and 2012/2013 before stabilizing for the rest of the decade with the exception of a peak in the number of cases detected in cattle in 2015/2016. Cases of chronic fasciolosis in sheep and fasciolosis in cattle peaked in 2012/2013.

**Fig. 2.**
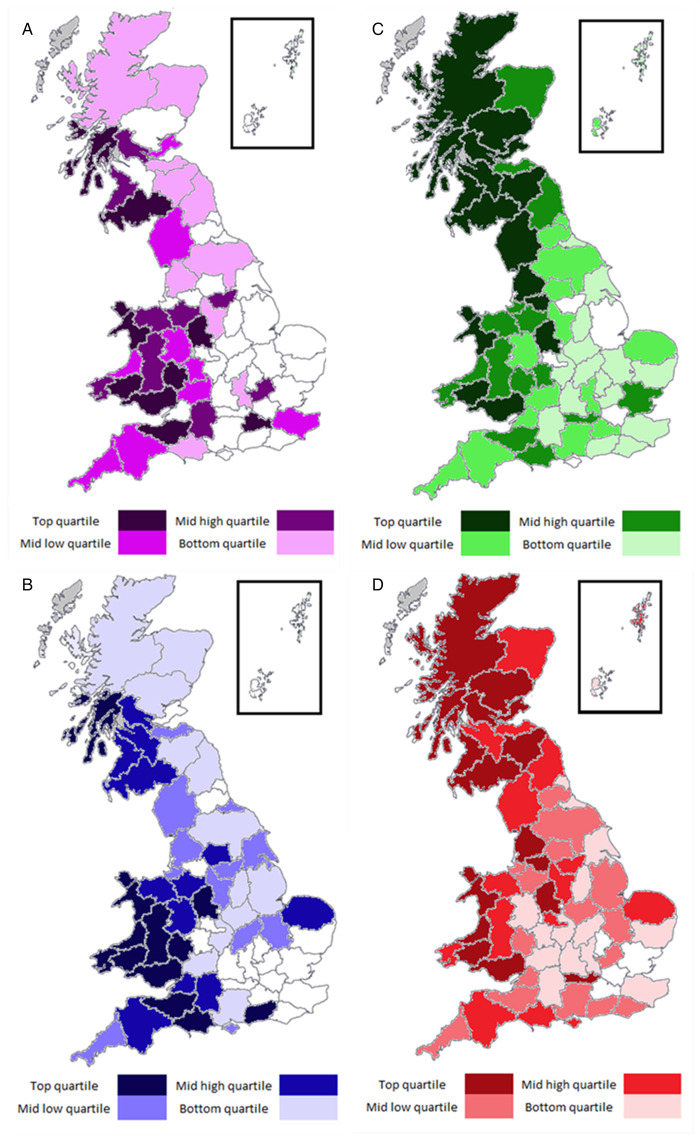
Distribution of VIDA diagnosis of fluke cases as a proportion of veterinary diagnostic submissions in APHA and SRUC surveillance counties in Great Britain between 2010 and 2019. A = rumen fluke sheep; B = rumen fluke – cattle; C = chronic fasciolosis – sheep; D = fasciolosis – cattle; white counties = no fluke cases diagnosed; grey counties = insufficient veterinary submissions.

Figure 2 shows the geographical distribution of VIDA diagnosis of rumen fluke and fasciolosis as a proportion of veterinary diagnostic submissions on average in each veterinary surveillance county in Great Britain between 2010 and 2019. A general trend observed sees fluke cases detected at higher rates in western counties of GB.

### Models

Table 2 presents the best fit model for the presence and number of rumen fluke VIDA diagnoses detected in sheep veterinary submissions and the associated significant variables. The absence of rumen fluke cases was significantly associated with lower average summer rainfall (*P* < 0.001) and lower average winter temperatures (*P* = 0.011). Higher rumen fluke cases were significantly associated with a higher proportion of sheep diagnostic cases positive for rumen fluke during the previous year (*P* < 0.001), whilst higher summer rainfall and winter temperatures were significantly associated with higher rumen fluke cases (*P* < 0.05). Counties with low sheep densities were significantly associated with lower rumen fluke cases in sheep compared to counties with high sheep densities (*P* = 0.001). The addition of the between-subject factors into the model reduced the model's adjusted ICC from 0.232 to 0.000.

**Table 2.** Zero-inflated negative binomial generalized linear mixed model of rumen fluke presence and prevalence based on sheep veterinary surveillance data from 60 APHA and SRUC surveillance areas between 2010 and 2019

Component	Variable	*B*	s.e.	*z*	*P*
Conditional Component	Intercept	−8.359	1.052	−7.949	<0.001
	Summer av. rainfall (9 year av.)	0.020	0.006	3.405	<0.001
	Winter av. temperature (9 year av.)	0.242	0.115	2.109	0.035
	Previous year rumen fluke cases in sheep	27.364	6.057	4.518	<0.001
	Low sheep density	−1.059	0.327	−3.243	0.001
	Medium sheep density	−0.230	0.245	−0.936	0.349
	High sheep density	0	–	–	–
Zero Inflated Component	Intercept	20.681	5.645	3.664	<0.001
	Winter av. temperature (9 year av.)	−3.267	0.900	−3.631	<0.001
	Summer av. rainfall	−0.057	0.023	−2.545	0.011

Table 3 presents the best-fit model for the presence and number of rumen fluke VIDA diagnoses detected in cattle veterinary submissions and the associated significant variables. The absence of rumen fluke cases was significantly associated with lower average summer rainfall (*P* = 0.015). Higher rumen fluke cases were significantly associated with a higher proportion of cattle diagnostic cases positive for rumen fluke during the previous year and higher summer sunshine hours and winter temperatures (*P* < 0.001). Higher summer rainfall was significantly associated with higher rumen fluke cases (*P* < 0.001). Counties with low and medium cattle densities were significantly associated with lower rumen fluke cases in cattle compared to counties with high cattle densities (*P* < 0.01). The addition of the between-subject factors into the model reduced the model's adjusted ICC from 0.152 to 0.07.

**Table 3.** Zero-inflated negative binomial generalized linear mixed model of rumen fluke presence and prevalence based on cattle veterinary surveillance data from 65 APHA and SRUC surveillance areas between 2010 and 2019

Component	Variable	*B*	s.e.	*z*	*P*
Conditional Component	Intercept	−11.709	1.203	−9.732	<0.001
	Summer av. rainfall (9 year av.)	0.031	0.006	4.977	<0.001
	Winter av. temperature	0.272	0.055	4.909	<0.001
	Summer av. sunshine hours	0.014	0.004	3.450	<0.001
	Previous year rumen fluke cases in cattle	25.942	6.170	4.205	<0.001
	Low cattle density	−1.132	0.305	−3.713	<0.001
	Medium cattle density	−0.772	0.273	−2.831	0.005
	High cattle density	0	–	–	–
Zero Inflated Component	Intercept	9.219	4.254	2.167	0.030
	Summer av. rainfall	−0.238	0.097	−2.446	0.015

Table 4 presents the best fit model for the presence and number of chronic fasciolosis VIDA diagnoses detected in sheep veterinary submissions and the associated significant variables. Higher chronic fasciolosis cases were significantly associated with a higher proportion of sheep diagnostic cases positive for chronic fasciolosis during the previous year and higher summer rainfall (*P* < 0.001). Higher summer raindays was significantly associated with higher chronic fasciolosis cases (*P* = 0.014). Counties with low sheep densities were significantly associated with lower chronic fasciolosis cases in sheep compared to counties with high sheep densities (*P* = 0.018). The zero-inflated component of those best-fit model did not contain any significant factors associated with the presence or absence of chronic fasciolosis cases detected in sheep veterinary submissions. The addition of the between-subject factors into the model reduced the model's adjusted ICC from 0.1 to 0.093.

**Table 4.** Negative binomial mixed model of chronic fasciolosis presence and prevalence based on sheep veterinary surveillance data from 60 APHA and SRUC surveillance areas between 2010 and 2019

Component	Variable	*B*	s.e.	*z*	*P*
Conditional Component	Intercept	−6.691	0.381	−17.580	<0.001
	Summer av. rainfall	0.020	0.002	11.255	<0.001
	Summer av. raindays (9 year av.)	0.015	0.006	2.454	0.014
	Previous year chronic fasciolosis cases in sheep	7.074	1.336	5.293	<0.001
	Low sheep density	−0.386	0.163	−2.362	0.018
	Medium sheep density	0.004	0.123	0.036	0.971
	High sheep density	0	–	–	–

Table 5 presents the best fit model for the presence and number of fasciolosis VIDA diagnoses detected in cattle veterinary submissions and the associated significant variables. The absence of fasciolosis cases between counties was significantly associated with lower average summer rainfall (*P* = 0.019). Higher fasciolosis cases were significantly associated with an increased proportion of cattle diagnostic cases positive for fasciolosis during the previous year and higher summer rainfall and lower summer temperature (*P* < 0.01). The addition of the between-subject factors into the model reduced the model's adjusted ICC from 0.092 to 0.019.

**Table 5.** Zero-inflated negative binomial mixed model of fasciolosis presence and prevalence based on cattle veterinary surveillance data from 65 APHA and SRUC surveillance areas between 2010 and 2019.

Component	Variable	*B*	s.e.	*z*	*P*
Conditional Component	Intercept	−4.018	0.523	−7.678	<0.001
	Summer av. rainfall	0.010	0.001	7.899	<0.001
	Summer av. temperature	−0.106	0.035	−2.980	0.003
	Previous year fasciolosis cases in cattle	17.749	1.734	10.235	<0.001
Zero Inflated Component	Intercept	28.957	12.666	2.286	0.022
	Summer av. rainfall (9 year av.)	−0.602	0.256	−2.355	0.019

Diagnostic testing of the residuals of each final model revealed no concerns regarding model fit. There were no significant deviations to the normality of scaled residuals, no significant deviations between the scaled residuals and transformed predictions and no zero inflation of residuals (*P* > 0.05) in any of the final models. There was no spatial or temporal autocorrelation of residuals (*P* > 0.05) in any model and the variance inflation factors (VIF) scores for each independent variable per final model were <2 indicating low collinearity (Lüdecke *et al*., 2021).

## Discussion

Rumen fluke has emerged as a prominent parasite in sheep flocks and cattle herds in Great Britain as well as multiple countries across Europe over the past decade. Despite a rapid emergence in the early 2010s (Huson *et al*., 2017), where case detection rates increased roughly five-fold between 2010 and 2014, case detection rates stabilized in cattle and decreased in sheep towards the end of the decade. Considering this, it is likely that rumen fluke is now well established across Great Britain (Bellet *et al*., 2016). A study by Jones *et al*. (2017*a*) found that ruminants on 61% of Welsh farms were infected with *C. daubneyi,* whilst surveys of individual cattle at slaughter have found rumen fluke prevalences of between 25 and 29% (Bellet *et al*., 2016; Sargison *et al*., 2016). Similar rapid rises in rumen fluke prevalence have been observed in France (Mage *et al*., 2002) and the Republic of Ireland (Toolan *et al*., 2015) amongst other countries. Yet it is still unclear what factors have driven these increases in *C. daubneyi* presence and prevalence, although it has been hypothesized that climate change (Naranjo-Lucena *et al*., 2018), decreased competition from *F. hepatica* to infect intermediate snail host *G. truncatula* (Mage *et al*., 2002), changes in anthelmintic classes commonly used to treat fasciolosis (Jones *et al*., 2017*a*) and high levels of animal movements (Sargison *et al*., 2019) may all have contributed.

It is apparent from the models created in this study, however, that climate is a key driver for rumen fluke prevalence, with rainfall, raindays, winter temperature and sunshine hours all identified as factors associated with rumen fluke prevalence. Considering this, a role for climate change in the parasite's establishment in western Europe is likely. Increased rumen fluke prevalence was observed in the western counties of Great Britain in this study, an epidemiological pattern similar to that of liver fluke. It is well established that the distribution and prevalence of liver fluke in the UK is strongly correlated with rainfall, with heavy summer rainfall increasing *G. truncatula* population size and geographical extent. Considering *C. daubneyi*'s reliance on *G. truncatula* as its intermediate snail host, a snail which thrives during wet summers, it is unsurprising that higher summer rainfall and/or raindays was significantly positively associated with rumen fluke presence and prevalence in both the cattle and sheep models. Modelling has demonstrated that the suitability of Great Britain's climate to *G. truncatula* has increased over the past 50 years (Fox *et al*., 2011), a factor which has likely been beneficial to *C. daubneyi*'s establishment. Mean summer sunshine hours was also a significant climate variable positively associated with rumen fluke prevalence in cattle. Sunshine hours has previously been found to be significantly associated with rumen fluke prevalence on farms in Wales (Jones *et al*., 2017*a*), and a biologically important role of light in stimulating *C. daubneyi* egg hatching has also been established (Chryssafidis *et al*., 2015). Considering that *C. daubneyi* will be in competition to infect *G. truncatula* with *F. hepatica* and potentially other trematode parasites of wildlife (Jones *et al*., 2015, 2017*b*), increased egg hatching rates may be vital to maximize lifecycle opportunities.

Another climate variable present within both rumen fluke models was average winter temperature, where rumen fluke prevalence was higher in sheep in surveillance areas with warmer winter temperatures and higher in cattle in years with warmer winter temperatures. Winter temperature is known to influence *G. truncatula* activity and survival. In mild winters, *G. truncatula* snails remain active for longer periods which may allow further development and shedding of infective larvae onto pasture (Relf *et al*., 2011). Mild winters also allow for a larger proportion of *G. truncatula* snails to successfully overwinter, potentially carrying over and shedding cercariae onto pasture the following spring (Fox *et al*., 2011). However, there was no significant association between winter temperature and fasciolosis presence or prevalence in equivalent models. Differences in fluke species pathogenicity and diagnostic methods applied to diagnose infections with each fluke species may have caused biases in the data set, meaning direct comparisons between liver and rumen fluke models can only be tentative. However, it is reasonable to hypothesize that the association between rumen fluke prevalence and winter temperature identified in this study may be an indication of a direct effect on *C. daubneyi* in the environment. Trematode metacercariae are highly vulnerable to sub-zero temperatures, with freezing known to make metacercariae unviable (Morley, 2015). The degree to which temperature influences *F. hepatica* metacercariae viability has been widely studied, with optimal survival rates present at temperatures between 1 and 5 °C (Turner *et al*., 2016). However, according to Boray and Enigk (1964), a considerable number of *F. hepatica* metacercariae can survive daily temperature fluctuations between −5° and 10 °C for at least 70 days and constant temperatures of −2 °C for at least 92 days. There is limited information regarding the viability of *C. daubneyi* or other paramphistome species’ metacercariae in relation to temperature. According to Chadhri and Gupta (1985), the viability of an unspecified *paramphistomum* sp. metacercariae was greater when stored at 25 °C for 3 days compared to when stored at 5 °C for this period. However, storage at 5 °C proved optimal for maintaining metacercariae viability for 12 or more days. The resilience of trematode metacercariae is known to vary depending on the environment in which the species evolved (Morley, 2015). For example, the metacercariae of philophthalmid eye flukes which evolved at higher latitudes have a greater tolerance of cold temperatures compared to those from lower latitudes (Nollen and Kanev, 1995), whilst the metacercariae of *Fasciola gigantica,* a trematode found in tropical and sub-tropical regions, has greater viability at 35°C compared to *F. hepatica* (Boray and Enigk, 1964). Considering that paramphistomes are historically associated with tropical and sub-tropical regions (Taylor *et al*., 2007), it is feasible that *C. daubneyi* may have evolved in non-temperate climates, which could explain a poorer tolerance to colder weather. However, uncertainty remains regarding the evolutionary and geographical history of *C. daubneyi.* Nevertheless, it is widely hypothesized that *C. daubneyi* was absent from temperate areas until recent decades (Sargison *et al*., 2016), and at present, there is no evidence in the scientific literature that *C. daubneyi* is prevalent in colder northern European countries such as Sweden (Huson *et al*., 2017) despite the presence of *G. truncatula* snails and *F. hepatica* (Novobilský *et al*., 2014). Milder winters have been observed in GB and western Europe over the past decades which has been associated to climate change (Kendon *et al*., 2021) and may have led to improved conditions for *C. daubneyi* to establish.

In each model, the previous year's fluke infection rate was significantly positively associated with that years’ fluke case rates. As the cases recorded in this dataset gives no indication of when infections occurred, it is feasible that at least some cases recorded would be carry over infections from the previous years. *F. hepatica* may survive in cattle and sheep for multiple years which supports this possibility, although treatment against *F. hepatica* is administered on 85% or more of UK farms according to recent surveys (Hoyle *et al*., 2021; Williams *et al*., 2021). Treatment against rumen fluke is not practised as routinely (Jones *et al*., 2017*a*; Hoyle *et al*., 2021), although there are some suggestions that the final host develops adaptive immunity against *C. daubneyi* which may limit its lifespan in ruminants (Atcheson *et al*., 2020). It is also likely that high fluke case rates would directly lead to increased new infection in the following year due to the nature of the fluke lifecycle. High levels of fluke eggs deposited on pasture due to high infection rates in livestock would lead to increased infection rates in intermediate snail host populations (Jones *et al*., 2017*b*) and ultimately higher prevalence in the final host the following year (Turner *et al*., 2016). Considering this, farmers are encouraged to limit pasture contamination through strategic anthelmintic treatment. The benefit of this strategy was demonstrated by Parr and Gray (2000), who observed limited *F. hepatica* infections in intermediate host snail populations and subsequently in livestock when animals were strategically treated to suppress pasture contamination compared to a control farm that did not. Furthermore, modelling suggests that treatment of animals in late winter and spring would on average lead to a reduction in fasciolosis risk the following summer by 65% (Beltrame *et al*., 2021). However, current guidance regarding the treatment of adult rumen fluke infections remains contentious, with treatment only recommended when clinical signs are observed alongside diagnosis when no other potential causes of ill thrift are identified (Forbes, 2018). This seems sensible considering the rarity of clinical paramphistomosis in temperate regions (Huson *et al*., 2017) and because of continued concerns on the sustainability of making widespread use of the limited compounds available to treat rumen fluke infections (Forbes, 2018). However, there is a risk that in certain years where conditions are optimal, heavy pasture contamination of rumen fluke eggs could translate into large infection burdens of the juvenile stages of the parasite in young cattle and sheep which can cause devastating losses (Anon, 2018; O'Shaughnessy *et al*., 2018). A potential solution would be to apply non-chemical methods to reduce pasture contamination and exposure of livestock to infective metacercariae such as drainage, fencing or rotational grazing, although the application of these non-chemical methods is often unattractive to farmers due to cost and practicability (Fairweather, 2011; Coyne *et al*., 2020). The models created in this study further highlights the importance of minimizing pasture contamination of fluke eggs, and additional consideration of optimal rumen fluke control strategies in livestock is needed as further data regarding rumen fluke pathogenicity, epidemiology and susceptibility to treatment emerges. The models also provide evidence that the incorporation of data regarding previous prevalence on a large geographical area, or potentially egg contamination levels on a farm or field level could strengthen disease prediction models.

In conclusion, rumen fluke is a highly prevalent parasite infecting cattle and sheep in Great Britain. Modelling revealed that rainfall, raindays, sunshine, winter temperature, the density of livestock and previous years case rates were significantly associated with rumen fluke prevalence across space and time. The associated factors identified in this study can form the basis of further epidemiological investigation of rumen fluke in Europe and future models of rumen fluke distribution and prevalence

## Data Availability

Data are available from the corresponding author upon reasonable request.
